# Illinois State Board of Health

**Published:** 1884-03

**Authors:** 


### Society Reports.

### ARTICLE VII.

REPORT OF PROCEEDINGS OF THE ILLINOIS STATE BOARD OF HEALTH. Quarterly meeting, Springfield, January 17–18, 1884.

The regular January quarterly—which is also the annual—meeting of the Illinois State Board of Health, was held in the rooms of the Board in the Capitol building, January 17 and 18, 1884.

Present at the Thursday (January 17) session: R. Ludlam, A. L. Clark, W. A. Haskell, A. W. H. Reen, and the Secretary, John H. Rauch.

On motion of Dr. Haskell, Dr. Ludlam was called to the chair in the absence of the President. Dr. Ludlam called the meeting to order, and presented Mr. Reen, of Peoria, the new appointee, vice Dr. McLean, whose term expired December 31, 1883.

The Secretary moved that the regular order be dispensed with, pending the arrival of the President, from whom a telegram had been received, announcing his delay at Vermont, Fulton county, on account of a disabled engine. The motion being carried, the Board went into executive session, during which, among other matters, the certificates of Drs. G. A. Allen and A. Jones were ordered revoked for unprofessional and dishonorable conduct, and the Secretary was instructed to notify several other parties to desist from practices deemed unprofessional and dishonorable by the Board, on pain of revocation of certificates. Upon charges filed with the Board, the Secretary was instructed to cite a number of others to appear at the next meeting, to show cause why they should not be debarred practice in Illinois on the ground of unprofessional and dishonorable conduct.

At the conclusion of the executive session, the Board proceeded to the election of officers for the ensuing year, the result being the re-election of the following gentlemen as their own successors:

The Hon. Newton Bateman, LL.D., President; John H. Rauch, M.D., Secretary; A. L. Clark, M.D., Treasurer.

The Chair also appointed Mr. Reen to serve on the auditing committee with Dr. Haskell.

The Treasurer, Dr. Clark, submitted his annual report, showing that, at the date of the last report, there was a balance of $220.91 in his hands; that $684 additional had been received through the Secretary's office during the year, and that $715.85 had been paid out on orders, leaving a balance of $189.06 at the close of the fiscal year, September 30, 1883.

On motion of Dr. Haskell, the report was received, and placed on file.

The auditing committee reported that it had examined and audited accounts, amounting to $2,306.57; had found the same to be correct, and recommended that they be paid.

The report of the committee was accepted, and at 10:45 P. M. the Board adjourned until Friday, January 18.

Friday, January 18, 10 o'clock A. M.—Present: Newton Bateman, W. A. Haskell, W. R. McKenzie, A. W. H. Reen, John H. Rauch. Under the regular order of business, the Secretary presented the following:

### QUARTERLY REPORT OF THE SECRETARY.

During the quarter ended Dec. 31, 1883, there have been received in the Secretary's office 930 communications, letters, reports, etc., exclusive of ninety-three diplomas submitted for verification, and the papers accompanying the applications for certificates in 142 cases. There were sent out 1,183 letters, postals, circulars and other communications, and about 8,000 copies of the publications of the Board. Seventy-nine telegrams were received, and 68 sent.

The office correspondence for the year amounts to 4,036 letters and other communications received, and 5,157 sent, exclusive of

562 diplomas submitted for verification, and the papers accompanying the applications for certificates from 787 physicians and midwives.

During the same period, 2,712 copies of Annual Reports and Official Registers, 585 copies of the advance sheets of the section on Medical Education, and 73,287 copies of Preventable Disease Circulars, Quarterly Reports and other printed matter, making an aggregate of 76,584 copies of publications, have been distributed.

CERTIFICATES AND LICENSES.

Certificates entitling to practice medicine under the Medical Practice Act, were issued to 86 graduates upon diplomas from legally chartered medical institutions in good standing, and 9 to practitioners on length of practice in the State; making in all 553 practitioners of medicine admitted to practice in Illinois during the year 1883. During the same period, 163 applications have been refused, and 11 certificates have been revoked; thus directly reducing the number of practitioners by 174, through the enforcement of the provisions of the Medical Practice Act.

Among the causes for refusal to issue certificates were the following: Presenting diplomas of institutions not recognized by the Board as in good standing; unsatisfactory personal or professional antecedents, habits or associations, warranting the charge of unprofessional and dishonorable conduct; intent to practice in an unprofessional and dishonorable manner, as by claiming to cure incurable maladies; to possess unusual skill, experience or facilities; and similar claims involving deceit and fraud upon the public. The revocations of certificates were in all cases based upon charges of unprofessional and dishonorable conduct.

Seven certificates were issued to midwives upon the credentials of recognized schools of midwifery, and four upon examination by the Board. A total of 51 midwives have thus been admitted to practice during the year—25 upon credentials, 7 upon length of practice in the State, and 19 upon examination. In all 40 presented themselves for examination, of whom 21 were rejected.

### SPECIAL MEETINGS.

The Board has held five special meetings during the quarter, namely, on Nov. 3 and 10, and on Dec. 1, 8 and 15. These meetings were of a purely executive nature, held for the purpose of taking action on sundry cases under the Medical Practice Act. At the meeting held on Saturday, Nov. 3, the certificate of Dr. Frank B. Smith, temporarily of Peoria, was revoked for unprofessional and dishonorable conduct.

Among the other business transacted, the cases of 11 practitioners, holding the certificates of the Board, and against whom various charges had been preferred, were satisfactorily adjusted, the offenders agreeing to refrain from the objectionable practices in the future. In a number of other cases, the parties have left the State, and the disposition of the charges against them should receive attention at the present meeting, as well also as the cases of some others cited to appear.

### MEDICAL COLLEGES.

The distribution of the advance sheets of the section on Medical Education and the Regulation of the Practice of Medicine, has resulted in developing a wider interest in the subject than was anticipated. Requests for copies have been received from every part of the United States and Canada, from England, East India and Australia. The section has been carefully revised and corrected, and, in accordance with the authorization of the Board, it is now being reprinted for sale by Mr. W. T. Keener, medical book publisher, of Chicago. The supply at the disposal of the Board is entirely inadequate to meet the demand.

Many questions have arisen during the quarter, concerning the new schedule of requirements of the Board in the matter of medical education. Most of these have been of such a nature as to admit of direct reply, but a number of others have been deferred for the consideration of the Board.

### SUITS AND OTHER PROCEEDINGS UNDER THE MEDICAL PRACTICE ACT.

Among the cases brought to the attention of the Board, through the Secretary's office, during the quarter, have been the following:

*Robertson.*—An "Indian doctor," F. O. Robertson, was arrested on the 26th of December, in Princeton, Bureau county, where he had appeared dnring the county fair in September, 1883, announcing his unusual skill and ability "to the accompaniment of evening concerts, rifle-shooting, stale jokes, etc., and claiming that his medicines are purely vegetable, free from morphine, quinine and all *other mineral* poisons." Five separate charges were made against him, and on the 28th of December, he pleaded guilty before the county court; fines were assessed against him on two charges, on payment of which, and costs, he was released under a promise to leave the locality.

Subsequent to his Princeton performances he turned up in Mascoutah, St. Clair county, where he managed to secure the aid of some of the German press in his behalf, and published his circulars and handbills in that language.

*Bogart.*—One T. D. Bogart, of Quaker Hill, Ind., was arrested in the early part of November, in Decatur, Macon county, as an advertising itinerant, making a specialty of the treatment of "chronic nasal catarrh and scrofulous sore eyes." He obtained a continuance of his case until December 7, and subsequently sent a petition to the Governor, purporting to be signed by many citizens of Hoopeston under his treatment for "chronic nasal catarrh, catarrhal fever, and scrofula in their many forms." The petitioners pray that the Governor "will grant him a permit by which he can treat cases as above set forth." The petition was referred to the Secretary, who replied to the petitioners that the Governor had no legal right to grant such a permit, and explained that the reason why Bogart was debarred from practice in Illinois was because of his failure to comply with the law.

*Bunce.*—A man, calling himself "Dr." A. W. Bunce, and claiming the benefit of the ten-year exemption clause, is also under arrest in Decatur, his claim that he practised ten years prior to July 1, 1877, being contradicted. It is asserted that he was digging wells for a livelihood about that time. It is proposed to give him an opportunity to prove his ten years' practice, or to demonstrate his fitness to practice medicine by undergoing an examination.

*Dunn.*—The advertisiug itinerant, E. C. Dunn, of Rockford, is complained of from Paxton, Ford county; Arcola, Douglas county; and Danville, Vermilion county. Like the preceding case, Bunce of Decatur, he is operating under the exemption clause. The question suggests itself whether such practice as he is engaged in is that contemplated by the statute, as entitling one to exemption from the necessity of making a prima facie show of fitness for the cure of the sick.

*Flowers.*—The notorious character, "H. D. Flowers, of Fulton City, Whiteside county," continues his rounds. Within a short time of each other complaints were received from his victims as far south and east as Ford county, and north and west as Jo Davies's.

*Chicago Quacks.*—In Chicago suits have been brought, mainly during the month of December, against Drs. G. J. Williams (also a "lawyer"), Lucas R. Williams (alias Dr. Lucas"), A. W. Boye, Wm. Clarke (son of F. D. Clarke, "no pay until better"), John Bate (alias "A. G. Olin"), John Kean ("no-cure-no-pay"), and a "Dr. Shroder" (a woman).

### THE PUBLIC HEALTH.

Scarlet fever has continued during the quarter in many localities, but without in any case assuming a serious epidemic form, while the death-rate from it remained unusually low. The German edition of the Board's circular on scarlet fever, alluded to in the last quarterly report as in preparation, has been published and distributed to many points. Diphtheria has also been reported from several places.

Small-pox has appeared at Stone Fort, Saline county; Farina, Fayette county; McLeansboro, Hamilton county; and Alton, Madison county. The disease was also reported, October 31, from Paris, Edgar county; but the case was subsequently ascertained to be chicken-pox. At Stone Fort the disease was introduced by a woman who had been attending the St. Louis fair, took sick a few days after her return, and died on the thirteenth day of an attack of unmodified, confluent small-pox. Of seven other members of her family, four contracted the disease, the remaining three escaping through successful vaccination after exposure. A relative's family, living in the adjoining township in Pope county, also contracted the disease, and in turn infected the attending physician. Except this latter and one woman, none of those attacked had ever been successfully vaccinated prior to exposure. The physician and woman above referred to had both been vaccinated in childhood, and escaped with mild attacks of varioloid. Of the remaining cases, seven in number, all died of unmodified confluent or hæmorrhagic small-pox. The situation became so serious, and so much apprehension existed in the community and neighborhood about the middle of November, that the Secretary was compelled to visit the locality in person, which he did November 20-24. Reporters, Drs. W. R. Osborne and D. Bozart, Stone Fort, attending physicians.—The case near Farina was also due to contagion contracted in St. Louis, an unvaccinated young woman, who finally recovered after a severe attack of hæmorrhagic small-pox. Fortunately the other members of the family in which the case occurred were all protected by vaccination, and no other cases resulted. Reporter, A. R. Hancock, M.D., Farina, attending physician.—On the first of December, an unvaccinated public scholar at McLeansboro was found in the febrile stage of what proved to be an attack of small-pox, from which, however, she ultimately recovered. Source of contagion unknown. A large number of others were exposed, and, although vaccination was freely resorted to, six more cases followed, of which number two died. None of the seven had ever been vaccinated prior to exposure. Reporter, C. M. Lyon, M.D., McLeansboro, attending physician.—A fatal case of small-pox occurred in Alton, during the early part of December, contracted in St. Louis. Notwithstanding the case was not discovered until the eleventh day, and numbers of persons were exposed in the boarding-house where it occured, only one other case resulted—one of his nurses, vaccinated in childhood, but not successfully revaccinated until after nearly two weeks' exposure. This patient died on the fifth day, the disease assuming the rare form of purpura variolosa. Reporter, W. A. Haskell, M.D., Alton, attending physician.

### TRICHINA.

Three outbreaks of trichiniasis—one of them resulting in three deaths—have occurred, the first during the early part of November, in a family near Gardner, Grundy county ; the second and third during December, in Woodburn, Macoupin county, and in Bloomington, respectively. The Gardner and Bloomington cases resulted from eating uncooked pork and sausage, and the Woodburn cases from raw smoked sausage. Specimens of the meat from Gardner and Woodburn were sent to the Secretary for examination, the character of the disease being only suspected, until the microscope showed, in one case, about 2,000, and in the other about 8,000 trichinæ to the cubic inch.

Dr. A. T. Darrah reports the Bloomington outbreak quite fully, and Drs. Taxis and Reid, of Gardner and Woodburn, respectively, have promised detailed reports of their cases. The matter, however, possesses little sanitary importance, except that it is well to put on record every authentic instance of the occurrence of trichiniasis and to determine its cause. Since 1866 I have noted every such instance in this State, and of the deaths resulting, which now number sixteen ; and without exception they have been caused by eating uncooked or imperfectly cooked pork in some form. I see no reason to modify the opinion expressed in 1881, in reply to an inquiry addressed to me by a committee of the Chicago Board of Trade and the State Department at Washington, to-wit: That, as a sanitarian, I regard the danger of human life from trichinæ as practically amounting to nothing, it being so easily prevented by thorough cooking.

The investigations which have been made during the past four years, both in this country and abroad, show American pork to be freer from trichinous infection than that of other countries. The hog in every country is subject to the parasite; even the wild swine in European forests have been found infected, and an outbreak of trichiniasis in 1881, at the village of Khiam, near the sources of the Jordan in Palestine, was caused by the flesh of a wild boar slain in the woods near the village. Prof. Virchow is recently reported to have condemned the outcry against American pork, as utterly illogical, unnecessary and unjustifiable by sanitary reasons, and adds that "no case of trichinæ in American hog meat has been proved to exist in Germany for ten years." Neither in Switzerland nor England has it been found necessary to interfere with the importation of American hog products; and yet in these countries their reputation is higher than ever before. It is only where the custom of eating raw pork obtains that severe or extensive outbreaks of trichinias occur. Such an outbreak was that at Emersleben, in Saxony, last fall. MM. Brouardel and Grancher, who made an investigation, found that it was not due to American pork at all, but that the trichinous hog, which was the origin of the disease, was born and reared in a stable at Emersleben, the offspring of an English boar and a native sow. Killed on the 12th of September, 1883, a slice of the carcase was eaten raw on the 13th by two men, who were taken ill on the 16th, and died a month later. Meantime, the rest of the meat, mixed with other, was minced up and sold between the 13th and 19th of September. Except by five persons, who cooked it a little, this minced meat was eaten perfectly raw, and within a short time there were two hundred and fifty persons sick, of whom forty-two died; and one hundred and twenty-six more cases, with eleven deaths, occured in neighboring villages among persons who ate sausage made by the same butcher. Trichinæ were found in abundance by MM. Brouardel and Grancher in the bodies of two of the victims. This is the most extensive outbreak that I know of; but as previously remarked, the question is one of economic and commercial importance, and in only a very limited sense has it any sanitary interest. Proper cooking will render the most badly infected piece of trichinous meat absolutely innocuous. Exposure to a temperature of 150 to 160 degrees, F., is fatal to the trichina.

PRECAUTIONS AGAINST DANGER TO LIFE FROM FIRES.

The recent loss of life by fire at Belleville suggests the point whether the Board can profitably take any further action looking to securing adequate provision for escape from, and for the extinguishing of, fires in public buildings. On different occasions within the past few years the Board has called attention to this subject, the last time in the fall of 1881, with reference to the danger from fire at seaside resorts, and this was emphasized, soon after the Secretary's circular-letter was sent out, by the destruction of no less than six of these inflammable structures within a very short time. Fortunately, these fires occurred at a season of the year when there were only a few, in some cases no inmates, and nothing worse than the rapid destruction of the buildings and contents happened. These events, however, serve to direct attention to the subject, and there is reason to believe that this class of buildings is much improved in this respect. I think it would be well to prepare a suituable letter with necessary instructions, and address to all those having charge of public buildings in the State, with the view of publishing a report on the condition of such structures, with reference to the number of stories, sizes and arrangement of rooms, number and dimensions of staircases, doorways, windows and other exits, special provisions for escape, and for extinguishing fires, etc. Such a report would serve a useful purpose in disclosing to what extent municipal, State, or other interference may be necessary to correct such dangers as are found to exist.

CHICAGO SEWAGE.

In my last qnarterly report, speaking of the solution of the various questions hinging upon the disposal of the sewage from the city of Chicago through the Illinois and Michigan canal, I observed that that it was "clearly the duty of the city of Chicago, immediately upon the close of navigation, to have the Bridgeport pumps put into operation, and their capacity and the capacity of the canal determined by actual experiment." I am glad to say that this experiment is now being carried on, and I am in receipt of a telegram from Superintendent Thomas, announcing that the "Bridgeport pumps are working finely, and are holding three feet of water in the canal above the level of the Chicago river."

Respectfully submitted,

JOHN H. RAUCH,

Secretary.

Dr. McKenzie moved that the report be accepted, and that the recommendations and actions of the Secretary be approved. The motion was adopted and the report was ordered to be printed in the account of the proceedings of the meeting.

The remainder of the session was devoted to executive business, and the discussion of charges against individuals and colleges, of which the Secretary submitted 42 various cases, not previously considered. Among other matters, then disposed of, it was ordered that diplomas issued by the College of Physicians and Surgeons of Joplin, Mo., and by the Kansas City Hospital Medical College for the current session, 1883–4, could not be received as the basis for certificates entitling to practice in Illinois.

Drs. Haskell and Rauch were appointed a committee to draft appropriate resolutions concerning the retiring member, Dr. McLean, and subsequently offered the following:

*Resolved*, That the members of the Illinois State Board of Health who have been associated with Dr. John McLean, of Pullman, during the past three years, part from him with unfeigned regret upon the termination o his period of service. His counsels have been characterized by prudence good sense, and moderation, and prompted by an earnest desire for the best interests of the public welfare, and for the elevation of the standard of the medical profession. His sterling qualities must command success for him in any position to which he may be called; and the best wishes of his fellow-members of this Board go with him.

On motion of Dr. Bateman, who left the chair for that purpose the resolution was unanimously adopted; and the Secretary was instructed to forward a copy of the same to Dr. McLean.

The Secretary submitted the following:

### STATEMENT OF EXPENDITURES OF THE ILLINOIS STATE BOARD OF HEALTH FOR THE FISCAL YEAR ENDING SEPTEMBER 30, 1883.

| | |
|---|---|
| Salary of Secretary | $2625 00 |
| Clerical services | 4276 00 |
| Traveling expenses of Board and Secretary | 1112 92 |
| Postage | 354 56 |
| Expressage | 160 75 |
| Telegrams | 89 35 |
| Stationery and Printing | 152 25 |
| Medical Journals, Books and Newspapers | 186 80 |
| Vaccine Virus | 35 00 |
| Sundries | 108 68 |
| Total | $9101 31 |

At four o'clock P. M., on motion of the Secretary, the Board adjourned the January meeting, *sine die.*

